# “Where There’s Smoke, There’s Fire”: A Content Analysis of Print and Web-Based News Media Reporting of the Philip Morris–Funded Foundation for a Smoke-Free World

**DOI:** 10.2196/14067

**Published:** 2019-06-06

**Authors:** Christina Watts, Becky Freeman

**Affiliations:** 1 School of Public Health Faculty of Medicine and Health The University of Sydney Camperdown Australia; 2 Cancer Council New South Wales Woolloomooloo Australia; 3 Charles Perkins Centre The University of Sydney Camperdown Australia

**Keywords:** tobacco industry, mass media, smoking, nontobacco products

## Abstract

**Background:**

In September 2017, the Foundation for a Smoke-Free World (FSFW), a not-for-profit organization with a core purpose “to accelerate global efforts to reduce deaths and harm from smoking” was launched. However, the legitimacy of the FSFW’s vision has been questioned by experts in tobacco control because of the organization’s only funding partner, Philip Morris International (PMI).

**Objective:**

This study aimed to examine the response to the FSFW in Web-based and print news media to understand how the FSFW and its funding partner, PMI, were framed.

**Methods:**

News articles published within a 6-month period after the FSFW was announced were downloaded via Google News and Factiva and coded for topic, framing argument, slant, mention of tobacco control policies, and direct quotes or position statements.

**Results:**

A total of 124 news articles were analyzed. The news coverage of the FSFW was framed by 6 key arguments. Over half of the news articles presented a framing argument in opposition to the FSFW (64/124, 51.6%). A further 20.2% (25/124) of articles framed the FSFW positively and 28.2% of articles (35/124) presented a neutral debate with no primary slant. The FSFW was presented as not credible because of the funding link to PMI in 29.0% (36/124) of articles and as a tactic to mislead and undermine effective tobacco control measures in 11.3% of articles (14/124). However, 12.9% of articles (16/124) argued that the FSFW or PMI is part of the solution to reducing the impact of tobacco use. Evidence-based tobacco control policies were mentioned positively in 66.9% (83/124) of news articles and 9.6% (12/124) of articles presented tobacco control policies negatively.

**Conclusions:**

The Web-based and print news media reporting of the formation of the FSFW and its mission and vision has primarily been framed by doubt, skepticism, and disapproval.

## Introduction

### Background

Tobacco smoking contributes, in epidemic proportions, to the global burden of disease, causing the death of approximately 7 million people each year [1]. In response to this, efforts to curb tobacco use have shown great success through the implementation of effective, evidence-based tobacco control policies. The establishment of the World Health Organization (WHO) Framework Convention on Tobacco Control (FCTC) in 2003 was a critical milestone for global tobacco control, when 168 countries signed the convention, signifying widespread international cooperation to accelerate the implementation of effective tobacco control measures globally [2]. As of January 2019, there are 181 parties to the convention, making the WHO FCTC not only 1 of the most widely adopted United Nations treaties [2] but also 1 of the most impactful because of its role in implementing and advancing tobacco control legislation and programs [3]. However, despite the success of the WHO FCTC, the global tobacco epidemic remains problematic, and the progress toward the implementation of effective tobacco control policies has been slow in many countries, particularly in low- and middle-income countries where 80% of the world’s smokers live [1].

On September 13, 2017, the former head of the WHO’s tobacco free initiative, Derek Yach, announced the establishment of the *Foundation for a Smoke-Free World* (FSFW), a not-for-profit organization with the mission to “accelerate global efforts to reduce deaths and harm from smoking, with the ultimate goal of eliminating smoking worldwide” [4]. In a commentary written by Derek Yach, he argues that there has been “complacency” in the adoption of WHO FCTC policies, and progress to reduce smoking is “lagging.” He calls for an increased “ambition” in tobacco control and proposes that harm reduction from smoking is the solution needed to achieve this change [5]. However, in setting out on the journey to achieve this mission, the FSFW has accepted nearly US $1 billion over 12 years from Philip Morris International (PMI) [5]. PMI has a long and documented history of opposing evidence-based tobacco control policies, and recent investigations have shown that PMI is continuing to promote its flagship cigarette brands to youth and young adults through advertising via social media influencers on Instagram and Facebook [6], in advertising campaigns such as “Be Marlboro” [7], and by handing out free cigarettes to young people at parties [8]. In 2016, PMI also lost a legal suit that PMI launched against the Government of Uruguay over the implementation of large graphic health warnings and a ban on misleading packaging [9]. These are just a small illustration of PMI’s most public actions to oppose tobacco control policies, which misalign with the FSFW’s stated ultimate goal of eliminating smoking worldwide.

In response, experts working in tobacco control and public health have largely questioned the legitimacy of the FSFW and Derek Yach’s motives and whether the FSFW has any role to play in the efforts to sustain and increase the reduction in tobacco smoking globally. In a statement following the announcement of FSFW, the WHO stated it will not partner with the FSFW and consequently called on governments and the public health community to reject any partnership with the FSFW [10]. In January 2018, the deans from 17 public health schools across the United States also released a statement announcing that they will not accept funding from the FSFW, prompting other universities, associations, and organizations to follow suit [11-13]. Tobacco control experts argued that the move by PMI to fund such an organization aligns with their longstanding agenda to support research for the purposes of promoting a positive public image and to regain influence within the public policy setting, whereas also publicly distracting the media from evidence-based tobacco control policies [14].

Although the tobacco control and public health community response to the launch of the FSFW has been primarily negative, how the FSFW is positioned in the news media has the potential to be heavily influenced and manipulated by both FSFW and PMI. The news media has the power to influence the general public’s knowledge and attitudes regarding health issues [15] and can even shape the health policy agenda [16]. The ways in which news events and issues are reported can influence how the target audience thinks, feels, and decides, and facts can be slanted to a particular side with tone, charisma, and rhetoric [17]. As PMI’s involvement with the FSFW aligns with their repeated efforts to rebuild a positive public reputation [18-20], it is likely that both PMI and FSFW have an interest in how the FSFW and affiliated research and programs are portrayed in the news media. At the heart of PMI’s objective to rebrand itself as a company dedicated to a “smoke-free future,” is an effort to shift public sentiment and ultimately, shape and influence the tobacco policy agenda [21]. It is, therefore, critical to monitor and examine how the global news media have responded to the launch of FSFW.

### Objectives

In this study, we examined how the global news media responded to the FSFW during a 6-month period following the announcement of the FSFW, to understand how the media framed the FSFW and its funding partner, PMI. We also documented any mention of proven, evidence-based tobacco control policies in the news reporting. All news actors were captured to build a picture of which key informants and messages are featured in the news coverage, as well as the influence of PMI and FSFW’s media releases in shaping the development of news articles.

## Methods

### Search Strategy

Google News and Dow Jones Factiva were used to search for Web-based and print news articles published within a 6-month period after the FSFW was publicly announced. The time period of data collection spanned from September 13, 2017 to March 13, 2018. Separate searches were conducted on both Google News and Factiva using the search terms “Foundation for a Smoke-Free/Smokefree World” and “Smoke-free/Smokefree” with “Foundation.” A further search was conducted on Factiva with the search term “Smoke-free/Smokefree” and PMI selected as the company reference. Searches conducted on Factiva were limited to the following Factiva source categories: newspapers, magazines and journals, and major news and business. Searches conducted on Google News specifically looked for print news articles and Web-based news websites (eg, Reuters, news.com.au, and The Guardian) but excluded blogs and letters.

**Table 1:** Coding variables.

Coding variable	Description
Topic	Overall, what is the news article about? (1 topic per article coded)
Framing argument	What is the argument presented in relation to the FSFW^a^? Framing argument was determined by identifying the argument that was presented most frequently within the articles
Slant	Is the article presenting the FSFW as a positive or negative initiative? Or is the article neutral toward the FSFW? Positive: defined by a framing argument that is in favor of the FSFW. Negative: defined by a framing argument that is opposed to the FSFW. Neutral: defined by no framing argument, but rather, neutral debate is presented
Mention of tobacco control policies	What tobacco control policies or initiatives are highlighted or mentioned in the article?
Direct quotes or position statements	Who is quoted or paraphrased in the article with a position or opinion on the FSFW?

^a^FSFW: Foundation for a Smoke-Free World.

The news articles that appeared in the search results were scanned for relevance. Any news article that did not make at least 1 reference to the FSFW was not downloaded for inclusion in the study. News articles obtained through Google News were downloaded using the Web-browser extension, NCapture, and were then imported into NVivo; a qualitative data analysis program from QSR International. News articles obtained through Factiva were downloaded and imported into NVivo. Exact duplicates of news articles were removed; however, news articles that were similar, but not exact replications, were retained.

### Content Analysis

All news articles were read in full by the first author, CW. News articles were coded and analyzed for topic, framing argument, overall slant, mention of tobacco control policies, and direct quotes or position statements (Table 1). Coding categories were developed iteratively following a similar methodological approach taken by Smith and Wakefield [22]. Each article was coded to 1 of 8 different topics, which represented the primary message of the article. Articles were also content analyzed and coded for the argument that framed the article, which was determined by identifying the argument that was presented most frequently within the article. Each of the framing arguments were categorized as either being in favor of, against, or neutral toward the FSFW. Articles were also content analyzed for references to tobacco control policies or initiatives and were coded in 11 categories. Direct quotes and position statements about the FSFW from individuals, associations, companies, or organizations were coded in 14 categories.

Finally, all news articles were also cross-checked against media releases from PMI and the FSFW that were issued within the data collection period. Media releases were sourced directly from the FSFW [23] and PMI websites [24], where all public media releases are published. Articles were coded as to whether the media releases were copied verbatim or were altered. This was determined by identifying articles published within 1 week of the media release date and systematically auditing articles for key quotations from the media releases. Articles that were written with the aid of a press release, but were not copied verbatim, were also coded for whether the article followed the same slant as the press release or not.

A random sample of 5 articles were selected and coded by an additional 4 coders, including the second author, using coding guidelines, to test inter-rater reliability. The inter-rater reliability was 93.5% (116/124).

## Results

### Overview

**Figure 1:**
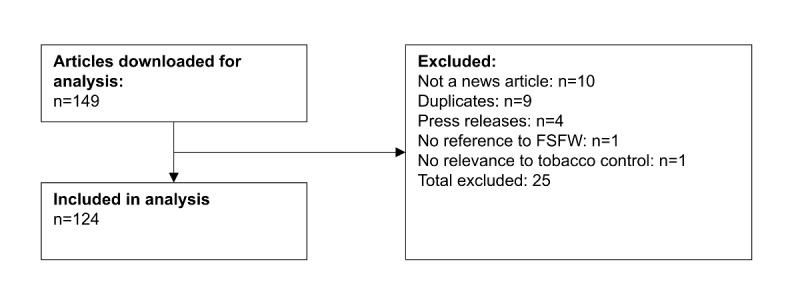
Flowchart for identification of articles to be included in analysis. FSFW: Foundation for a Smoke-Free World.

A total of 149 news articles were downloaded from Google News and Factiva. A total of 9 news articles were exact duplicates and were removed from the sample. Upon reading each article in full, a further 4 articles were found to be media releases, 1 article did not make any reference to the FSFW, 1 had no relevance to tobacco control, and 10 articles were news bulletins or less than a paragraph in length, and so were also excluded from the study (Figure 1). A total of 124 news articles were included in the final content analysis. A total of 4 of these articles were published interviews with André Calantzopoulos (PMI chief executive officer), Derek Yach (FSFW president), Michael Bloomberg (WHO global ambassador for noncommunicable diseases and Bloomberg Philanthropies founder), and Allan Erickson (team leader of National Tobacco Reform Initiative).

### Topics and Overall Slant

Articles that reported on PMI wanting a smoke-free future or PMI’s strategy to achieve a smoke-free future was the most frequent article topic, with 24.2% (30/124) of all articles coded under this topic. A further 23.4% (29/124) of articles were about individuals or entities refusing funding or any affiliation with the FSFW, and 21.8% (27/124) of articles explored and questioned whether the vision of the FSFW is plausible or not.

When cross-tabulated with the overall slant of each article, whether that be positive, negative, or neutral toward the FSFW, it was found that among articles with an overall positive slant (25/124, 20.2%), articles written about PMI wanting a smoke-free future were most frequent (13/124, 10.5%). Among articles with an overall negative slant toward FSFW (64/124, 51.6%), articles written about individuals or entities refusing funding or any affiliation with the FSFW were most frequent (21/124, 16.9%), followed by articles that explored and questioned the plausibility of the vision of the FSFW (14/124, 11.3%). The results of the article topics by slant are summarized in Table 2.

**Table 2:** Article themes by overall slant.

Topic	Brief description	Slant, n (%)			
		Positive toward the FSFW^a^	Negative toward the FSFW	Neutral toward the FSFW	Total
Bloomberg STOP Foundation announced	Articles that report on the announcement or establishment of the STOP Foundation, funded by Bloomberg Philanthropies.	0 (0.0)	7 (5.6)	1 (0.8)	8 (6.5)
FSFW announced	Articles that report on the announcement or establishment of the FSFW.	1 (0.8)	0 (0.0)	4 (3.2)	5 (4.0)
New grants, research, and programs from the FSFW	Articles that report on any new grants, research projects, or programs that are funded from the FSFW.	4 (3.2)	2 (1.6)	0 (0.0)	6 (4.8)
PMI^b^ wants a smoke-free world	Articles that report on PMI wanting a smoke-free future or world and its future investment strategy to achieve this.	13 (10.5)	8 (6.5)	9 (7.3)	30 (24.2)
Reduced-risk products (RRP)	Articles that report on the regulation of RRPs or the need for new avenues in tobacco control and the potential of RRPs fulfilling this need.	3 (2.4)	0 (0.0)	4 (3.2)	7 (5.6)
Refusing funding or affiliation with the FSFW	Articles about individuals or entities refusing funding or any affiliation with the FSFW.	1 (0.8)	21 (16.9)	7 (5.6)	29 (23.4)
The plausibility of the vision of the FSFW	Articles that explore/question whether the vision of the FSFW is plausible or not. Articles within this category generally explore perspectives on the FSFW specifically to uncover whether it can be trusted or not.	3 (2.4)	14 (11.3)	10 (8.1)	27 (21.8)
Tobacco industry interference	Articles about how the tobacco industry is interfering or the ways in which the tobacco industry continues to interfere in the development and implementation of effective tobacco control policies.	0 (0.0)	12 (9.7)	0 (0.0)	12 (9.7)
Total		25 (20.2)	64 (51.6)	35 (28.2)	—^c^

^a^FSFW: Foundation for a Smoke-Free World.

^b^PMI: Philip Morris International.

^c^Not applicable.

**Table 3:** Framing arguments presented.

Framing argument		Articles by frame, n (%)
**Argument in support of FSFW^a^**		25 (20.2)
	PMI^b^ is funding the FSFW in an effort to ensure sustained profitability of the tobacco industry.	9 (7.3)
	The FSFW or PMI is part of the solution to reducing the impact of tobacco use.	16 (12.9)
**Argument in opposition to FSFW**		64 (51.6)
	FSFW is not credible simply because of funding link to PMI	36 (29.0)
	PMI is funding the FSFW to disingenuously makeover their image	10 (8.1)
	The FSFW is a tactic to ensure sustained profitability of the tobacco industry.	4 (3.2)
	The FSFW is a tactic to mislead and undermine effective tobacco control measures	14 (11.3)
Balanced debate		35 (28.2)

^a^FSFW: Foundation for a Smoke-Free World.

^b^PMI: Philip Morris International.

### Framing Arguments

The news coverage of the FSFW was framed by 6 key arguments, as can be seen in Table 3. Over half of the news articles presented an overall framing argument that was classified as against the FSFW (64/124, 51.6%), whereas arguments in favor of the FSFW made up 20.2% (25/124). A further 28.2% of articles (35/124) presented a neutral debate with no primary framing argument. The most frequent framing argument presented was that the FSFW is not credible because of the funding link to PMI (36/124, 29.0%). Statements questioning the credibility of the FSFW included:

It is the height of hypocrisy for PMI to proclaim that it is helping solve the tobacco problem while it aggressively markets cigarettes. [25]

Philip Morris isn’t the solution to the tobacco problem. Philip Morris is the cause. [26]

Articles also argued that the FSFW was established as a tactic to mislead and undermine effective tobacco control measures (14/124, 11.3%), to deceitfully ensure sustained profitability of the tobacco industry (4/124, 3.2%), and that PMI is funding the FSFW to disingenuously makeover their corporate image (10/124, 8.1%). Articles that presented arguments in favor of the FSFW most frequently argued that the FSFW or PMI is part of the solution to reducing the impact of tobacco use (16/124, 12.9%). A further 7.3% of articles (9/124) argued that PMI’s decision to fund the FSFW is a logical and genuine effort to ensure the industry sustains profitability in the future through investment in reduced-risk products. For example:

Yet Philip Morris International seems sincere in wanting to have people stop smoking combustible cigarettes—to go smoke-free—and switch over to e-cigs as an alternative. [27]

### Tobacco Control Policies and Measures Mentioned

Evidence-based tobacco control policies were commonly referenced or mentioned within the news articles analyzed. The WHO FCTC was mentioned most frequently across all articles (31/124, 25.0%), with the majority of articles presenting the WHO FCTC in a positive or neutral light (14/124, 11.3% and 15/124, 12.1% of the total number of articles, respectively). Tobacco excise was also frequently mentioned in articles (26/124, 20.8%), along with advertising and promotion bans (22/124, 17.7%) and graphic health warnings (21/124, 16.9%). Overall, 66.9% (83/124) of all articles mentioned tobacco control policies or measures positively, whereas only 9.6% (12/124) of articles presented tobacco control policies or measures negatively. A full list of tobacco control policies and measures referenced in the articles can be seen in Table 4.

**Table 4:** Tobacco control policies and measures referenced in news articles (% of total number of articles).

Tobacco control policies mentioned^a^	Positive^b^, n (%)	Neutral^c^, n (%)	Negative^d^, n (%)	Total, n (%)
WHO FCTC^e^	14 (11.3)	15 (12.1)	2 (1.6)	31 (25.0)
Tobacco excise	17 (13.7)	7 (5.6)	2 (1.6)	26 (20.8)
Advertising and promotion bans	17 (13.6)	4 (3.2)	1 (0.8)	22 (17.7)
Graphic health warnings	16 (12.9)	4 (3.2)	1 (0.8)	21 (16.9)
Smoke-free environment policies	7 (5.6)	5 (4.0)	2 (1.6)	14 (11.3)
Plain packaging	5 (4.0)	6 (4.8)	0 (0.0)	11 (8.9)
Low-nicotine cigarettes	0 (0.0)	10 (8.1)	0 (0.0)	10 (8.1)
Smoking cessation support	4 (3.2)	2 (1.6)	3 (2.4)	9 (7.2)
Point of sale restrictions	2 (1.6)	2 (1.6)	0 (0.0)	4 (3.2)
Mass media campaigns	0 (0.0)	1 (0.8)	1 (0.8)	2 (1.6)
Single cigarette sales ban	1 (0.8)	0 (0.0)	0 (0.0)	1 (0.8)
Total	83 (66.9)	56 (46.5)	12 (9.6)	

^a^If multiple policies were mentioned within 1 article, they were coded separately so the total exceeds the number of articles.

^b^Policy mentioned is framed as being effective, proven, or evidence-based.

^c^Policy mentioned with no statement of effectiveness or whether or not the policy is proven or evidence-based.

^d^Policy mentioned is framed as being ineffective or not having adequate effect on reducing smoking.

^e^WHO FCTC: World Health Organization Framework Convention on Tobacco Control.

### Quotes or Position Statements

Articles were coded for quotes and position statements found within the articles. Quotes from tobacco companies were most frequently paraphrased in all articles, with 43.5% (54/124) of all articles having at least 1 quote or statement from a tobacco company. Although the majority of these were quotes from PMI (49/124, 39.2%), which described the FSFW and PMI’s vision for a smoke-free world as positive, a small fraction of quotes was from other tobacco companies (6/124, 4.8%):

One senior executive at a rival manufacturer praised PMI for scoring a “huge PR coup”, but claimed that the Swiss-based company was not doing anything markedly different from its competitors. “It has said it will go smoke-free “as soon as possible’, although it has not put a time frame on achieving it,” he said. “I think you'll see PMI selling Marlboro cigarettes for many years to come. [28]

Unidentified tobacco company executive

Both the FSFW and public health groups, associations, and organizations were each quoted or had their position statement paraphrased in approximately 40.3% (50/124) of all articles (38.7% and 40.0%, respectively). Derek Yach was the primary spokesperson for the FSFW, with 38 of the total of 48 articles that quoted or paraphrased the FSFW, specifically quoting Yach. A total of 1 article was a published interview with Yach. The next most frequent organization or individual quoted or paraphrased was the United Nations, including the WHO, with 37.6% (47/124) of all articles including statements or quotes from the WHO. All statements attributed to the WHO were opposed to the formation and funding of FSFW. Health academics were also quoted in 27.2% (34/124) of the articles; however, they were slightly more varied in their position on the FSFW. Of the 23 health academics quoted, 3 were in favor of the FSFW, 18 were against, and 2 held neutral positions. Examples of positive and negative quotes or statements from health academics include:

Another anti-smoking expert and Adjunct Professor at the Centre for Health Law, Policy & Ethics at University of Ottawa, David Sweanor, is on the same page as Yach. He is inviting everyone at the Tobacco or Health Conference to consider a different, and perhaps more effective approach to the usual “let’s wrestle tobacco companies to the ground” kind of reasoning. [29]

The idea of taking money that's from the tobacco industry is just antithetical to everything we do, Karen Emmons, Dean for academic affairs at Harvard’s public health school. [30]

### Media Releases

News articles are frequently generated as a direct result of media releases, which can be reproduced verbatim as a published news article or rewritten with quotes or content from the media release paraphrased with at times, a different overall slant or angle. During the data collection period, there were 4 media releases from the FSFW and 1 from PMI that were included in at least 1 article. A total of 3 news articles in total published the media release verbatim with no changes, 3 used quotes from the media releases but kept the same slant and angle as the media release, and 10 articles had quotes from the media releases, though the overall slant and angle of the article was different or conflicting with that of the media release (Table 5).

**Table 5:** Article reliance on media releases from Philip Morris International (PMI) and Foundation for a Smoke-Free World (FSFW).

Media release title	Organization	Media release reproduced verbatim (n=3), n	Quote from media release, different angle (n=10), n	Quote from media release, same angle (n=3), n
Global foundation launches to accelerate an end to smoking	FSFW	0	1	1
Letter to the World Health Organization	FSFW	0	5	1
Public invited to shape Foundation for a Smoke-Free World’s US $1 billion research agenda	FSFW	1	1	0
Accelerating an end to smoking	FSFW	1	0	0
Philip Morris International announces support for the establishment of the Foundation for a Smoke-Free World	PMI	1	3	1

## Discussion

### Principal Findings

The global news media have reported the announcement of the FSFW; however, the portrayal of the foundation and its mission and vision has primarily been framed by doubt, skepticism, and disapproval. A majority of articles were slanted negatively toward the FSFW, and the most frequent framing argument presented was that the FSFW was not credible because of the funding link to PMI. Other arguments against the FSFW were that the foundation was established as a tactic to mislead and undermine effective tobacco control measures, to deceitfully ensure sustained profitability of the tobacco industry, and to disingenuously makeover PMI’s corporate image. Encouragingly, at least 1 evidence-based tobacco control measure was mentioned or referenced positively within a majority of the news articles analyzed.

Despite attempts by both PMI and the FSFW to steer media coverage in a positive direction, the tobacco control community’s views opposing this new venture dominated the news reporting. This is a positive finding for public health advocates as it is indicative that tobacco control policies and perspectives are generally accepted as reputable, newsworthy, and fundamental in the efforts to reducing the burden of smoking. News media have repeatedly shown to be influential in communicating health messages to the public [31-34], and news editors are fundamental in determining what stories are published and how these issues are framed. Given that 66.9% of all articles (83/124) made mention of evidence-based tobacco control policies positively and just 9.6% of articles (12/124) referred to evidence-based tobacco control policies negatively, it appears that the decades of effort to raise public support for tobacco control policies has influenced news editors’ and journalists’ perceptions of tobacco issues and solutions and overall acceptance of public health viewpoints. This aligns with previous research, which found that newspaper editors’ largely expressed support for tobacco control measures and objectives in tobacco-related editorials [22]. Given the high dependency of politicians and key decision makers on the media and how issues are framed, there is therefore potential for a widespread rejection of the FSFW among governments, policy makers, and the public if news reporting continues to align with the viewpoints of the tobacco control community [35].

The ways in which issues are reported on in the media can also be heavily impacted by the content of media releases. Corporate strategies for engagement with the media are seen to be essential to shape a company’s reputation and build respectability and credibility [35]. However, inaccuracies or embellishments in original media releases can easily be reproduced as fact in news media articles [36,37]. As PMI has attempted to rebuild its public reputation through public relations and social media strategies [18-20], it was essential to examine whether the media releases from PMI and the FSFW resulted in additional news articles and whether or not these news articles were reproduced verbatim or published with the same or a conflicting angle. The 4 media releases from the FSFW and 1 from PMI did not result in a large response in Web-based and print news media, with only 3 articles reproduced verbatim from the media release. Although 10 articles had used the messaging from the media releases, the resulting articles were not framed in support of FSFW.

In what was clearly an attempt to secure positive news coverage, we noted that an Australian journalist traveled to Japan as a guest of PMI. This was revealed in a concluding disclosure statement within the news article. The article written by this journalist presented a balanced debate about PMI’s involvement in reduced-risk products, citing quotes from reputable evidence-based organizations such as the WHO and Cancer Council Australia, alongside quotes from the manager of PMI in Japan [38]. Another journalist wrote of his experience going to the PMI headquarters in Switzerland and interviewing PMI’s chief executive, André Calantzopoulos, which may indicate that the trip was paid for by PMI [39]. The subsequent news article that was published by this journalist argued that PMI is part of the solution to reducing tobacco related disease. It is possible that other journalists received similar benefits without public disclosure, which may have had an impact upon the way the way in which PMI and the FSFW was framed in the final news articles.

Although the findings of this study broadly indicate positive results for the public health and tobacco control agenda, the FSFW and PMI were still able to deliver their key messages and have key media spokespeople quotes within a significant proportion of all news articles (44.0% and 38.7%, respectively). The most frequent topic presented in the news articles was PMI’s key public relations message that it is working for a smoke-free future, irrespective of the overall slant of the article. The presentation of some supportive views from health academics in the media may also create confusion and a perception of “in-fighting” and disunity among experts in the field.

Given PMI’s deceptive history and false attempts to publicly rebrand itself as a socially responsible company committed to a smoke-free future, it is essential that the tobacco control community unite to reinforce why the FSFW cannot be considered a reputable research-focused not-for-profit organization. The FSFW lacks independence from PMI, as revealed from a review of the FSFW governing documents showing that PMI’s funding is contingent on the FSFW working toward its stated goals, which are aligned with PMI’s agenda. The FSFW also lacks transparency and accountability, and board members, who were appointed by the FSFW president, are paid US $50,000 per year, which is far beyond the standard of other nongovernment organizations [40]. In recent months, Derek Yach wrote an open letter on behalf of the FSFW inviting the WHO executive board to work with the FSFW [41], which subsequently resulted in the Global Centre for Good Governance in Tobacco Control coordinating a response from 120 organizations and 182 individuals calling on the WHO to reject the FSFW’s bid to partner together to reduce the impact of smoking [42]. Such coordinated efforts are key to minimizing the influence of FSFW and PMI in tobacco control research and policy making into the future. It is also critical that the news media is continually monitored to ensure that the tobacco industry and associated organizations, such as the FSFW, are not successfully undermining public health efforts to reduce tobacco use. This is critically important as the FSFW begins to roll out funding and grants to public health researchers, as has happened in New Zealand with the creation of the *Centre of Research Excellence on Indigenous Sovereignty & Smoking* [43].

### Limitations

News articles were not able to be analyzed for readership numbers, nor for the number of times each article was shared on social media; therefore, the overall reach of the articles was not determined. The study also focused on Web-based and print news articles only and excluded other Web-based channels that are used to obtain and share information, such as social media. As the data were obtained through Google News and Factiva, there is also no guarantee that all Web-based and print news articles were included in the analysis.

### Conclusions

In the 6 months following the announcement of the FSFW, the global news media primarily portrayed the FSFW and PMI with doubt, skepticism, and disapproval, despite efforts to shape the development of news articles through corporate media releases and media spokespeople. Although this is a positive finding for the public health agenda, it is essential that the tobacco control community continue to provide a unified voice in the media on this issue to minimize the influence of the FSFW and PMI in tobacco control research and policy development.

## Abbreviations

FCTC: Framework Convention on Tobacco Control
FSFW: Foundation for a Smoke-Free World
PMI: Philip Morris International
WHO: World Health Organization
